# Causes and consequences of intraspecific variation in animal responses to anthropogenic noise

**DOI:** 10.1093/beheco/arz114

**Published:** 2019-07-01

**Authors:** Harry R Harding, Timothy A C Gordon, Emma Eastcott, Stephen D Simpson, Andrew N Radford

**Affiliations:** 1 School of Biological Sciences, University of Bristol, Bristol, UK; 2 Marine Scotland Science, Aberdeen, UK; 3 Biosciences, College of Life & Environmental Sciences, University of Exeter, Exeter, UK; 4 Australian Institute of Marine Science, Perth, WA, Australia

**Keywords:** biological responses, environmental stressors, experiments, management, mitigation, noise pollution

## Abstract

Anthropogenic noise is a recognized global pollutant, affecting a wide range of nonhuman animals. However, most research considers only whether noise pollution has an impact, ignoring that individuals within a species or population exhibit substantial variation in responses to stress. Here, we first outline how intrinsic characteristics (e.g., body size, condition, sex, and personality) and extrinsic factors (e.g., environmental context, repeated exposure, prior experience, and multiple stressors) can affect responses to environmental stressors. We then present the results of a systematic search of the anthropogenic-noise literature, identifying articles that investigated intraspecific variation in the responses of nonhuman animals to noise. This reveals that fewer than 10% of articles (51 of 589) examining impacts of noise test experimentally for intraspecific variation in responses; of those that do, more than 75% report significant effects. We assess these existing studies to determine the current scope of research and findings to-date, and to provide suggestions for good practice in the design, implementation, and reporting of robust experiments in this field. We close by explaining how understanding intraspecific variation in responses to anthropogenic noise is crucial for improving how we manage captive animals, monitor wild populations, model species responses, and mitigate effects of noise pollution on wildlife. Our aim is to stimulate greater knowledge and more effective management of the harmful consequences of this global pollutant.

## Teaser

To understand fully how noise pollution affects animals, we must consider differences between individuals. Less than 10% of published experimental studies on noise effects investigate variation due either to individual characteristics (such as size and sex) or external factors (such as current context or previous exposure). We explain how greater consideration of individual variation is vital for understanding man-made noise as a global pollutant and for developing mitigation strategies to protect animals.

## INTRODUCTION

Human population growth, rapid urbanization and infrastructure development, greater resource exploration and extraction, and the expansion of transportation networks have all contributed to the increased production of anthropogenic noise, altering terrestrial and aquatic soundscapes worldwide (Kight and Swaddle 2011; Shannon et al. 2015; Buxton et al. 2017). Many human activities generate noise within the hearing ranges of other animals, at sound levels above those found naturally and with different acoustic characteristics from abiotic and biotic sounds (Hildebrand 2009). Those man-made additions to the acoustic environment that contain little or no useful information and which have negative consequences on wildlife represent a well-recognized form of pollution. A wide variety of anthropogenic noise sources have been shown to affect invertebrate, fish, amphibian, bird, and mammal behavior (e.g., disrupting vocal communication, foraging, antipredator responses, and parental care), physiology (e.g., causing stress, hearing damage, and immune-system impairment), and development (e.g., reducing growth and causing morphological malformations), with resulting fitness consequences (for recent reviews, see Morley et al. 2014; Shannon et al. 2015; Kunc et al. 2016). However, most research has only considered whether noise pollution has an effect and the nature of its impact. Typically, empirical studies are inherently based on the assumption that conspecifics are ecologically equivalent, reporting responses as a mean cohort effect. Such a simplification ignores intraspecific (within-species) variation (Radford et al. 2016a).

Considerable variation exists between individuals of the same species for both intrinsic and extrinsic reasons, causing differences in the way that conspecifics look, behave, and respond to natural selection pressures, such as predation risk, food availability, and novel environments (Bolnick et al. 2003). It is therefore inevitable that when presented with anthropogenic stressors, individuals from the same species will respond in different ways (Bolnick et al. 2011). These varied responses may define the difference between success and failure; the likelihood of mortality or the ability to emigrate, to adapt through genetic changes, or to respond via phenotypic plasticity (Engås et al. 1996; Höglund et al. 2008; Cripps et al. 2014). Intraspecific variation in responses can also have far-reaching impacts on the population dynamics, community structure and ecosystem function of entire groups of animals (Post et al. 2008; Rudman et al. 2015; Charette and Derry 2016; Des Roches et al. 2017). Indeed, in some cases intraspecific variation can have a greater influence than interspecific differences on overall community responses to environmental change (Crutsinger et al. 2006; Siefert and Ritchie 2016; Raffard et al. 2019). Furthermore, varied responses set the stage for future evolution, as the cohort of individuals capable of reproducing following an anthropogenic stress event defines the evolutionary potential of the postdisturbance population (Medina et al. 2007; Bijlsma and Loeschcke 2012). To consider only “mean” responses to anthropogenic stressors is therefore to underappreciate the likely consequences of the disturbance; a lack of population-level impacts may be masking more subtle but important within-population changes. Conversely, consideration of intraspecific variation facilitates a more comprehensive understanding of the impacts of anthropogenic stressors on animals, the likely consequences for wider ecosystems, and the best management strategies to address these changes.

In this review, we begin by outlining the existence and importance of intraspecific variation in response to environmental stressors, and why its consideration with respect to anthropogenic noise is needed. We explain how variation arising from intrinsic characteristics (e.g., body size, body condition, sex, and personality) and extrinsic factors (e.g., environmental context, repeated exposure, prior experience, and multiple stressors) can affect responses in biological systems, and the consequences of such variation. We then report on a systematic review of the literature relating to the impacts of anthropogenic noise on nonhuman animals. We provide a comprehensive list of experimental studies that have investigated intraspecific variation in responses to noise, and offer qualitative and quantitative summaries of the scope and findings of that research. Moreover, we draw on an assessment of those existing studies when making suggestions for best practice in designing and implementing robust experimental research that would benefit the field moving forwards. Finally, we explain how a greater focus on intraspecific variation in response to anthropogenic noise is crucial for improving the management of animals in captivity, monitoring the impacts on wild populations, modeling species responses, and mitigating the effects on wildlife. Our aim is to stimulate a greater understanding of the importance of intraspecific variation when determining both the impacts of anthropogenic noise and how best to mitigate this global pollutant.

## THE EXISTENCE AND IMPORTANCE OF INTRASPECIFIC VARIATION

Intraspecific variation is caused by a range of intrinsic characteristics and extrinsic factors. Fundamentally, genotypic and epigenetic differences between individuals underpin intrinsic phenotypic characteristics (e.g., body size, body condition, sex, and personality) that vary within a population (Skinner 2015). Individuals with different characteristics may respond differently to environmental and anthropogenic stressors in terms of both their behavior and physiology (as discussed in detail below). These responses may be the consequence of, or be mediated by, differences in life-history trade-offs and strategies (Stearns 1997; Zera and Harshman 2001). Considerable intraspecific differences also arise due to extrinsic factors, including variation in the environmental context in which a stressor is experienced, repeated exposure to or prior experience of a stressor, and the presence and magnitude of multiple stressors. Many of these extrinsic factors (such as repeated exposure or prior experience) are likely underpinned by the flexibility in behavioral and/or physiological responses to sustained exposure to stressors; phenotypic plasticity is often the first line of defense when organisms are confronted with environmental change (Chevin et al. 2010; Wong and Candolin 2015). In this section, we describe several general mechanisms by which intraspecific variation can exert an influence on responses to environmental stressors and highlight the importance of considering this variation for an understanding of responses to anthropogenic disturbances such as noise. We do not provide an exhaustive list of potential characteristics and factors, but use illustrative examples where there is strong existing evidence for an influence.

### Intrinsic characteristics

**Figure 1:**
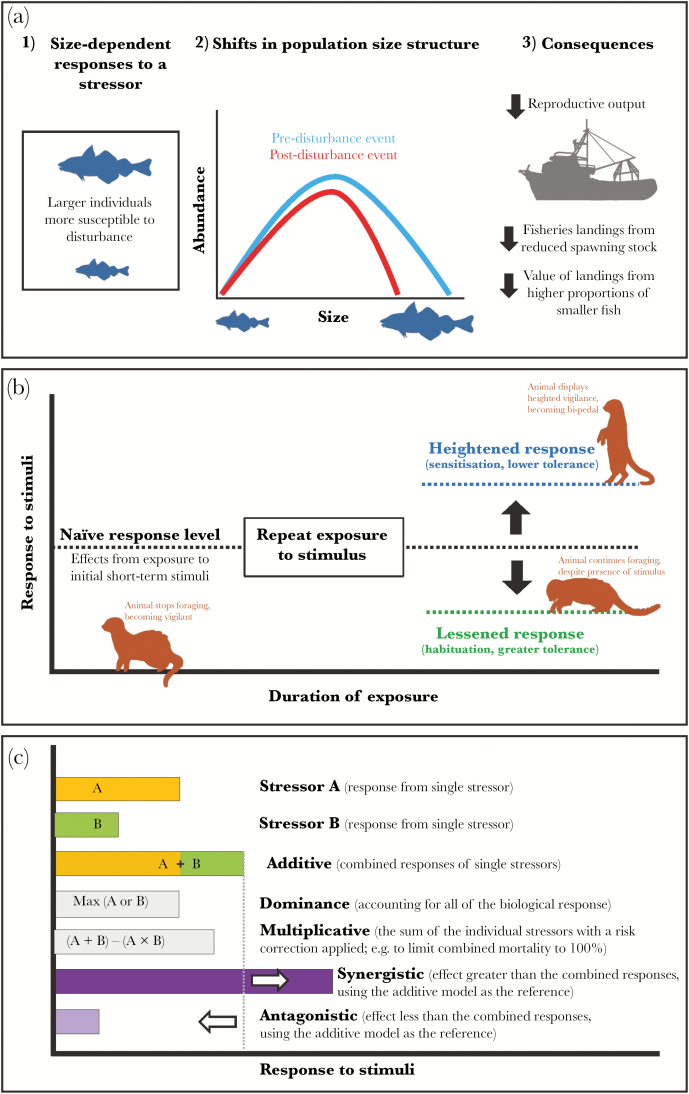
Importance of considering intraspecific variation in responses to environmental change. (a) Body size can affect responses to environmental stress with potential consequences for population-size structure and reproductive output, and thus implications for human economics and food security. (b) Prior experience of a stimulus may lead to a change in the level of response exhibited. (c) Multiple stressors can result in a variety of different response levels depending how the individual stressors interact (Côté et al. 2016). Images in figure drawn by WiseArt.net.

Variation in body size, which often scales with age (Sebens 1987), affects responses to environmental stressors due to fundamental differences in physiological mechanisms, morphology, and behavior (Spear et al. 1996; Ortiz-Santaliestra et al. 2006; Pörtner and Knust 2007). Size-dependent selection can drive changes in the demographic structure of populations (Figure 1a); for example, selective harvesting of larger individuals by commercial fishing fleets can cause shifts toward higher proportions of younger age classes among the spawning stock (Ottersen et al. 2006). In many taxa, body size correlates with fecundity (Shine 1988) and so stressors that truncate population size-structure may impact population reproductive potential and recruitment success, while also reducing the capacity to cope with further stress in variable environments (Kiffney and Clements 1996; Walther et al. 2002; Planque et al. 2010).

Conspecific individuals differ in body condition as a result of the heterogeneous nature of food availability and from inherent variation in routine metabolic rates (Metcalfe and Monaghan 2001; Killen et al. 2011). Variation in body condition results in considerable differences in behavioral and physiological functioning (Duckworth et al. 2001), and can therefore affect responses to environmental stress. Animals in poor condition with low energy reserves may display more risk-prone behaviors (Caraco et al. 1990), be unable to maintain optimal physiological functioning when challenged by a stressor, or fail to recover from additional environmental stresses (Sokolova et al. 2012; Sokolova 2013). Considering variation in body condition is important for the development of management and mitigation strategies to alleviate anthropogenic stress. For instance, during periods of reduced foraging opportunities or increased physiological stress, animals may be more at risk from anthropogenic stressors, and thus mitigation strategies become increasingly important at these times.

Sex-dependent effects occur in animals due to differences in morphology, biochemical processes, and hormonal profiles (McClellan-Green et al. 2007; Palanza 2017). For example, if there are sex differences in baseline levels of stress-induced hormones, which influence individual responses to disturbances (Pottinger et al. 1996; Dalla et al. 2011), then males and females may respond differently to the same environmental stressor. In some species, sex-mediated responses to stress may be an adaptive mechanism associated with different energetic requirements (Afonso et al. 2003). Different effects of environmental stress on each sex may have profound population-level consequences, including with respect to sex ratios which may become altered by, for instance, sex-specific mortality (Grüebler et al. 2008) or impacts on temperature-dependent sex determination (Parrott and Blunt 2005; Jensen et al. 2018). Potential impacts will be species-specific and related to mating strategy, but could include a decline in reproductive output affecting overall population viability (White et al. 2017; Jensen et al. 2018).

Intraspecific variation in animal personality—defined by a suite of behavioral traits consistent across time and environmental context—has been shown in multiple taxa (Sih et al. 2004; Réale et al. 2007). Personality covaries with physiological and neuroendocrinological mechanisms, determining an individual’s coping style; that is, how they deal behaviorally and physiologically with environmental stress (Carere et al. 2010). Personality type can affect how individuals perform in changing environments: in some systems, bold, fast-exploring, proactive individuals may do well in less-risky, stable environments, whereas slow-exploring, reactive behavioral types may perform better in high-risk environments and in situations of environmental change as they may have greater behavioral flexibility (Guillette et al. 2011; Sih et al. 2012). Maintenance of variation is important for adaptability to future environmental fluctuations (Dall et al. 2004; Sih et al. 2012). Furthermore, intraspecific variation in personality can have important implications for ecological processes, with variation in predator personalities shown to influence the composition of prey communities (Royauté and Pruitt 2015).

### Extrinsic factors

The current environmental context, including food availability and predation risk, can affect behavior exhibited by individual organisms (Lima and Dill 1990; Sih et al. 2004). Behavioral variability due to different environmental contexts reflects a trade-off between the risk from a stressor and the benefit gained from continuing a current activity (Lima and Dill 1990). For instance, many animals reduce their foraging during periods of increased predation risk (Clarke 1983; Lima and Dill 1990), and anthropogenic disturbances can influence predation risk (Chalfoun et al. 2002). Treating context as homogenous across studies compromises the quality of documented information about predicted responses to environmental stress.

Behavioral and physiological responses can change with repeated exposure to stressors, across a range of taxa (Figure 1b) (Burger and Gochfeld 1999; Bejder et al. 2009). These modifications can also be transferred to offspring through epigenetic mechanisms (Dias and Ressler 2014). Moreover, anthropogenic stressors vary across time and space (Hildebrand 2009; Mekonnen and Hoekstra 2015), meaning that individuals within a population or in different populations are likely to experience different conditions from one another. Variation in this prior experience can influence current responses, resulting in stronger effects due to sensitization, or weaker effects due to increased tolerance or habituation (Bejder et al. 2009). Using data based on short-term responses from assays that do not link directly to fitness may therefore under- or over-estimate realized impacts on populations (Bejder et al. 2009).

Organisms are rarely exposed to stressors in isolation, due to the multitude of anthropogenic threats faced by animals worldwide; these threats include light and chemical pollution, changing climates, hypoxia, acidification of marine and freshwater systems, and habitat destruction and fragmentation (Millennium Ecosystem Assessment 2005; McBryan et al. 2013). The effects of multiple stressors can be additive or multiplicative, or one stressor can dominate another; additionally, interactions may be synergistic or antagonistic (Figure 1c) (Côté et al. 2016; Gunderson et al. 2016). Understanding responses to single stressors does not always allow realistic predictions of responses to multiple stressors (Darling and Côté 2008); populations may show no adverse effects to particular pollutants in isolation, but the addition of another stressor may cause a markedly different response (Relyea and Mills 2001) or even stress individuals beyond their physiological limit (Fasola et al. 2015).

## STATE OF KNOWLEDGE WITH RESPECT TO ANTHROPOGENIC NOISE

We performed a systematic search of the peer-reviewed literature that has investigated the impacts of anthropogenic noise on nonhuman animals (see Supplementary Material for methods), with three main aims. First, we identified the number and scope of studies examining intraspecific variation in response to anthropogenic noise. Second, we used the resulting comprehensive list of experimental studies to compare findings relating to different intrinsic characteristics and extrinsic factors. Finally, we drew on an assessment of those existing studies to make suggestions for best practice in the design and implementation of experimental research that would benefit the field moving forwards.

### Research focus to-date

**Figure 2:**
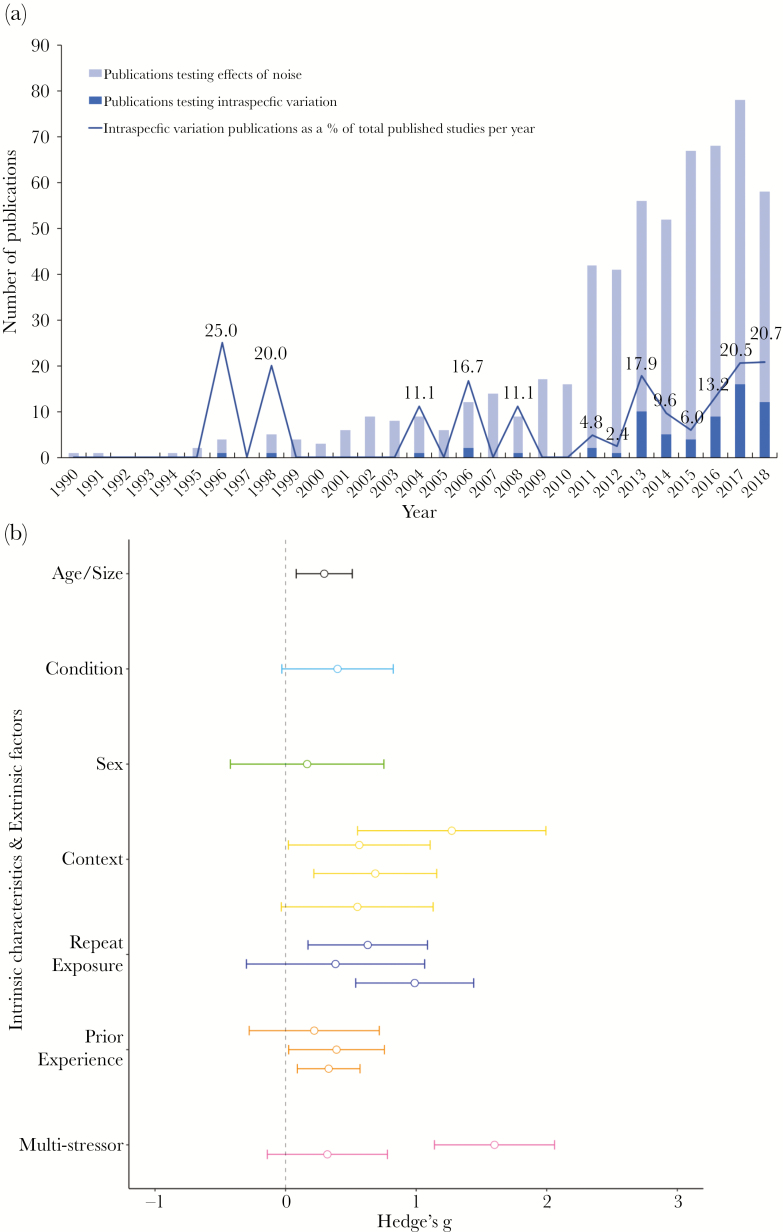
(a) Number of peer-reviewed publications per year that have investigated the effects of anthropogenic noise, and intraspecific response variation, in nonhuman animals. (b) Standardized effect sizes (Hedge’s *g*) for experimental studies in Supplementary Table S1 (calculated where possible and using illustrative examples where studies present more than one response metric) for different sources of intraspecific variation. Points and associated error bars represent composite standardized effect sizes (CES) and associated 95% confidence intervals from individual studies. CES were calculated in the following way: the weight of each group per study (1/variance of the effect size) was determined, and subsequently multiplied by the individual effect size (ES × weight); the composite effect size was then determined by dividing the sum of the effect size × weight by the sum of the weights. 95% confidence intervals were calculated with the following equation $\left[CI=CES+1.96\;\left(\sqrt{\left(1/\sum weights\right)}\right)\right]$. All CES are presented as positive integers to enable illustrative comparisons across studies.

The body of literature investigating the effects of anthropogenic noise on nonhuman animals has increased rapidly in the last decade (Figure 2a; Shannon et al. 2015). The proportion of peer-reviewed studies considering intraspecific variation has also been growing, especially since 2013, although the absolute number still remains low (Figure 2a). It is possible (as in all research fields) that a publication bias exists toward articles that find an effect; there may be some that set out to test for intraspecific variation but, on finding no evidence, subsequently pooled results to report a general effect of noise. From our literature search, we identified 65 articles that have tested intraspecific variation in response to anthropogenic noise. These comprise 51 experimental studies (detailed in Supplementary Table S1) and 14 observational studies (Supplementary Table S2), representing 8.7% and 2.4%, respectively of all articles published on anthropogenic noise that met our criteria. At present, the majority of noise studies testing intraspecific variation have considered extrinsic factors (*n* = 41, 71%), of which *repeated exposure* (*n* = 16) has been examined the most, followed by *environmental context* (*n* = 13)*, prior experience* (*n* = 8), and *multiple stressors* (*n* = 4). Intrinsic characteristics are less well-represented in our identified articles (*n* = 17; 29%); where an article documents consideration of two or more sources of intraspecific variation (*n* = 7), they are included multiple times in this assessment. Among intrinsic characteristics, *sex* (*n* = 8) has been investigated the most often, followed by *body size/age* (*n* = 6), *body condition* (*n* = 2), and *personality* (*n* = 1).

The experimental studies conducted to-date span a broad taxonomic range and have considered a variety of response measures. *Fish* are the most well-documented taxa (*n* = 22, 42% of experimental studies investigating intraspecific variation), followed by *birds* (*n* = 14, 27%), *mammals* (*n* = 8, 15%; aquatic: *n* = 5, terrestrial: *n* = 3), *arthropods* (*n* = 5, 10%; aquatic: *n* = 3, terrestrial: *n* = 2), and *amphibians* (*n* = 3, 6%); one study considered both *amphibians and arthropods*. Shannon et al. (2015) identified birds and marine mammals as by far the most-studied taxa in terms of noise impacts in general; the relative preponderance of fish studies on intraspecific variation likely reflects a recent upsurge in their consideration in anthropogenic-noise research (Kunc et al. 2016). In general, there is a strong taxonomic bias toward vertebrates, despite invertebrates making up 97% of known animals, having great ecosystem and commercial importance, and offering the opportunity for valuable experimental tractability (Morley et al. 2014). With regards to specific response measures, the majority of experimental noise studies considering intraspecific variation have focused solely on *behavioral* responses (*n* = 37, 73%), compared with eight (16%) for *physiological* measures and six (12%) where *both* behavior and physiology data have been collected. Only two studies (4%) have directly measured *fitness* impacts; while fitness estimates are often logistically more challenging to determine, they are what is ultimately required to assess population consequences.

### Current knowledge base

There are some qualitative differences in findings between different sources of intraspecific variation in the experimental work conducted to-date; any conclusions drawn at this stage need to be cautious, due to the small number of relevant studies in each case. Of the 58 measured aspects of intraspecific variation detailed in Supplementary Table S1, 44 (76%) are reported as having a significant effect on the response to anthropogenic noise. Overall, intrinsic characteristics (13 out of 17 cases; 76%) and extrinsic factors (31 out of 41; 76%) were equally likely to have a significant influence. However, at the level of specific characteristics and factors, there were considerable differences. All studies considering *body size* (*n* = 6) and *personality* (*n* = 1) reported significant effects on responses to noise, with *environmental context* and *repeated exposure* having a significant influence in 92% and 81% of studies, respectively. There is greater variation between studies considering each of *sex* (63%), *prior experience* (63%), and *body condition* (50%) as aspects of intraspecific variation. Only 25% of studies investigating *multiple stressors* reported an alteration in the effect of anthropogenic noise in the presence of an additional stressor.

We also considered, where possible, the standardized and composite effect sizes of intraspecific variation found in experimental anthropogenic-noise studies (Supplementary Table S1). Only rarely were the effect sizes for individual categories of interest or the intraspecific variation itself reported. We therefore attempted to calculate effect sizes ourselves, but this proved difficult due to a lack of relevant information. In the future, it would be useful if studies either included the means, standard errors/deviations, and sample sizes or the raw data to allow for accurate calculation of effect sizes from more complex experimental designs (repeated measures), or if they reported explicitly the statistical tests comparing the categories in question; only 29% (15/51) of studies in Supplementary Table S1 provided even some of this information. Consideration of the composite effect sizes we were able to calculate (*n* = 15) for different intrinsic characteristics and extrinsic factors indicated that no particular source of intraspecific variation causes an obviously greater magnitude in response differences to noise than any other (Figure 2b). A large composite effect size represents either a single characteristic (e.g., male or female) that is substantially more sensitive to noise than its equivalent opposite, or two or more characteristics that are affected by noise compared with the baseline/control conditions. In the four sources of intraspecific variation for which composite effect sizes could be calculated for more than one study, only one (*multiple stressors*) shows no overlap in confidence intervals, suggesting a substantial difference in response between studies. The different effect sizes for the two studies assessing multiple stressor impacts of noise may be related to the use of different response metrics (behavioral and physiological), which has been shown to affect the overall magnitude in response across the anthropogenic noise literature (Cox et al. 2018). From the remaining three sources, all three (*prior experience*, *repeated exposure,* and *context*) comprised studies where effect sizes overlapped with each other. Clearly, formal meta-analytic comparisons will only become possible with a greater number of suitable studies in the future.

### Future experimental studies

To improve our understanding of intraspecific variation in responses to noise, more and robust experimental tests are required. We suggest that a series of key decisions can aid the design and implementation of such tests; these are not mutually exclusive. Some of these decisions are broadly applicable to most, if not all, research fields and are frequently discussed. For instance, consideration of the relative advantages and disadvantages of captive versus field-based work, the need for suitable controls and sample sizes, and avoidance of pseudoreplication. We outline the importance of these fundamental concepts in Supplementary Table S3, highlighting existing examples of good practice from experimental articles on intraspecific variation in noise responses. Decisions that have more specific relevance to the study of anthropogenic noise are described in detail below.

Rigorous anthropogenic-noise research needs to involve suitable acoustic measurements, including consideration of what is known about the hearing thresholds of the study species. Full characterization of the sound field is required (rather than just the reporting of single decibel values) and should be presented in the appropriate domain for the species in question (Francis and Barber 2013; McKenna et al. 2016; Nedelec et al. 2016). For aquatic studies on fish and invertebrates, this includes reporting acoustic metrics in both particle-motion and sound-pressure domains (Nedelec et al. 2016); for terrestrial studies, the correct frequency weighting for the taxa needs to be applied (McKenna et al. 2016). Of studies in Supplementary Table S1, only 62% clearly do this (11 out of 25 fish and aquatic invertebrate studies; 17 out of 20 terrestrial studies). It has been suggested that sound measurements be recorded with both a Z-frequency weighting (flat response) and a weighting more appropriate for the study species (Francis and Barber 2013); only two examples of this approach exist in Supplementary Table S1 (LaZerte et al. 2016, 2017). Determining the best sound-characterization approach should ideally be informed by knowledge of the hearing range of the species being studied; in some species at least, there can be ontogenetic changes in hearing thresholds (Kenyon 1996; Wright et al. 2011). However, care needs to be taken when using published hearing thresholds; for example, the validity of many published fish-hearing measurements has recently been called into question (Hawkins et al. 2015).

In addition to clear and detailed reporting of acoustic metrics, it is important to consider the advantages and disadvantages of using loudspeaker playback (the methodology employed in 43 of the studies in Supplementary Table S1) versus real noise sources (nine studies in Supplementary Table S1) for experiments on the impacts of anthropogenic noise. Loudspeaker playback enables isolation of noise as the stressor, free from visual disturbances and other potential confounds (e.g., wake effects from passing boats or ships). However, use of loudspeakers results in sound fields that can differ considerably from those in real-world situations (Okumura et al. 2002; Quadros et al. 2014; Slabbekoorn 2016). Additionally, loudspeakers tend to provide only a point sound-source which is unrealistic for many types of anthropogenic noise, including vehicle traffic; this can be overcome with the use of multiple loudspeakers to create, for example, a phantom road (McClure et al. 2013). To provide acoustic validity, real-noise sources are required but this presents a number of challenges. First, it is often logistically difficult to obtain access to real noise sources. Second, robust experimental design requires multiple exemplars of the noise source to minimize pseudoreplication (Kroodsma et al. 2001). The latter is particularly problematic when testing for impacts of noise in aquatic environments (e.g., commercial shipping or pile-driving), however motorboats offer the opportunity for well-controlled experiments (Simpson et al. 2016). While captive experiments are constrained to using loudspeaker playback, large-scale mesocosm and field-based studies allow the potential use of both acoustic methods, and therefore facilitate a beneficial comparison. Using both methods allows a demonstration of the effect of an actual human activity while also assessing the importance of the noise component alone, but there is only one study in Supplementary Table S1 that combines the use of both loudspeaker playback and real noise sources (Harding et al. 2018).

Most published work investigating intraspecific variation in anthropogenic-noise effects has focused on either behavioral and/or physiological responses (see “Research focus to-date”). So, the range of response measures could be profitably broadened, especially given the rapid advancement in genetic-sequencing technologies and reductions in processing costs (Connon et al. 2018). It is increasingly possible to use methods such as restriction site-associated DNA sequencing to explore genotypic variation (Paris et al. 2015), and transcriptomics to reveal the genetic effects of stressors (Pespeni et al. 2013), as well as determine potential plasticity and evolutionary adaptability (Munday et al. 2013); the use of these methods is in its infancy in anthropogenic-noise studies (see Chen et al. 2018 for an example). Ultimately, though, a full understanding of population and ecosystem consequences will require consideration of impacts on individual fitness. Ideally studies would measure these directly rather than extrapolating from, for instance, short-term behavioral and physiological responses. Clearly measuring fitness is logistically challenging, but Casper et al. (2013) and Potvin and Macdougall-Shackleton (2015) have demonstrated that it can be feasible with respect to intraspecific variation in response to anthropogenic noise.

## APPLICATIONS FOR CAPTIVE AND WILD ANIMAL POPULATIONS

We believe that improving current understanding of intraspecific variation in responses to anthropogenic noise will increase our capability to manage captive animals effectively, monitor impacts on wild populations, model species responses, and mitigate the effects of noise pollution. Below, we provide specific suggestions as to how and why increasing our understanding can be valuable in each of these areas.

### Management

Incorporating intraspecific variation in noise responses into the management of captive animals could provide benefits to welfare, food productivity, and experimental control in research studies. Captive systems are often inherently noisy (e.g., transportation and construction noise in zoos raise ambient sound levels, as do pumps and aerators in aquaculture facilities; Bart et al. 2001; Shepherdson et al. 2004), but can potentially be quieted by noise-reduction techniques (Davidson et al. 2007). Elevated noise in captivity can cause stress and negatively affect growth, condition, and survival (Banner and Hyatt 1973; Shepherdson et al. 2004; Anderson et al. 2011). However, the effects of noise exposure may differ between cohorts or life-stages; for instance, animals may be especially vulnerable early in life (Davidson et al. 2007, 2009; de Soto et al. 2013; Nedelec et al. 2014). It is widely accepted that the feeding regimes needed to meet the energy and nutrient requirements of growing animals will change with age, and so diets are prepared accordingly for captive animals (Gardner et al. 1988; Wecke and Liebert 2013). If there are age-specific responses to noise, then tailoring noise-reduction management techniques to particular life-stages may similarly be beneficial to animal welfare, growth rates, and productivity; in aquaculture, this could arise through, for instance, a better feed-conversion efficiency (Davidson et al. 2007). Moreover, acoustic noise in captive research systems should be minimized to remove unwanted statistical variation, potential confounding factors and possible biases in response data (Sabet et al. 2016). Importantly, if intraspecific variation in response to noise exists, individuals from more or less susceptible cohorts should be split evenly between different experimental treatments to avoid biases.

### Monitoring

Considering intraspecific variation is crucial when monitoring the responses of wild animal populations to anthropogenic noise. If intrinsic characteristics cause undetected or unconsidered response variation, then population assessments and predictions about resilience to environmental stress may be misleading (Mimura et al. 2017). For instance, European eels (*Anguilla anguilla*) in poorer body condition were shown to be more affected than those in better condition by ship noise (Purser et al. 2016). Depending on when impact assessments are made, noise responses may appear more or less severe as a result of temporal fluctuations in body condition within populations (Brosset et al. 2015). Studies focusing on population averages may also provide inaccurate impact assessments when responses to noise change with repeated exposure or prior experience (Radford et al. 2016b; Harding et al. 2018). Monitoring populations near to and far from human activities would allow cohorts with varying degrees of noise exposure to be assessed (Harding et al. 2018), but in doing so it is important to control for habitat type and other anthropogenic disturbances, to avoid potential confounds and isolate noise as the stressor (Francis et al. 2012). Additionally, incorporating long-term acoustic-monitoring data, such as those from the Ocean Noise Reference Station Network (Haver et al. 2018), into environmental impact assessments (EIAs) could allow for consideration of prior experience and how exposure to different noise types can lead to specific or generalized response changes (Radford et al. 2016b). Including context-based and cumulative-impact evaluations into EIAs and monitoring could further reduce uncertainty when predicting behavioral and fitness effects (Ellison et al. 2012; Nowacek et al. 2015; Harris et al. 2018).

Interactions between species are highly flexible and can be influenced by intraspecific variation (Pruitt et al. 2012; Lichtenstein et al. 2016). For example, variation in the levels of an aggressive phenotype in a population of *Anelosimus studiosus* spiders was shown to alter relationships with heterospecifics and affect reproductive performance (Pruitt et al. 2012). Incorporating intraspecific variation of this nature may prove important for accurately determining the impact of noise on interactions between species—both antagonistic/conflict relationships (Simpson et al. 2016; Nedelec et al. 2017b) and those of a more cooperative or mutualistic nature (Nedelec et al. 2017a)—and at the community level (Moran et al. 2016). In a hypothetical example, whilst a given predator–prey relationship may appear to be affected by noise in favor of one party (Simpson et al. 2016), repeated exposure may adjust the balance of the relationship if, say, the prey becomes more tolerant and the predator continues to display its original response. Identifying the potential impacts of anthropogenic noise on the functional diversity of community assemblages (type, range, and abundance of organismal traits in a community; Díaz et al. 2007) will develop greater insight into how fundamental ecological processes and ecosystem stability may be affected. Predictions about impacts on functional diversity would be improved by considering intraspecific variation (Cianciaruso et al. 2009) because there can be differences in functional roles depending on such intrinsic characteristics as size and age (Bonaldo and Bellwood 2008). Intraspecific variation can have greater effects on indirect ecological interactions than removal or replacement of a species (Des Roches et al. 2017), and influence the strength of trophic cascades in a community (Post et al. 2008).

### Modeling

Modeling species responses to anthropogenic noise would likely be improved by inclusion of intraspecific variation. Predictive modeling can be used for EIAs and for projecting future species distributions and understanding functional and community-level changes (Chevin et al. 2010; Rossington et al. 2013). Empirical investigations of physiological and behavioral responses are valuable for parameterizing both population-persistence models and individual- and trait-based models, and for interpreting presence/absence and abundance data in combination with environmental variables in species-distribution models (SDMs) (Chevin et al. 2010; Koenigstein et al. 2016). Failure to capture intraspecific variation in baseline parameters may artificially reduce model uncertainty, but will also reduce the value of predictions when deciding mitigation measures and developing management strategies. Mechanistic population models can include multiple size/age classes and variation in phenotypic traits, while individual-based models can be expanded to include genotype (Moran et al. 2016). For example, including tree growth data as a measure of intraspecific variation improved the quality of pine tree distribution models relative to models that treated all trees as equal, and produced different future predictions as a result (Oney et al. 2013). Indeed, incorporating intraspecific variation into SDMs in this manner has been shown to alter predicted responses of species to environmental change in several cases (Valladares et al. 2014), and is therefore likely also to be important when modeling responses to anthropogenic noise.

### Mitigation

Strategies for mitigating the impacts of anthropogenic noise should include the identification of traits that make particular individuals within a population especially vulnerable. For example, male natterjack toads (*Epidalea calamita*) exhibit high site-fidelity during breeding seasons, as they remain located at a particular pond from which they call to attract free-ranging females (Sinsch 1992). In a hypothetical local noise-pollution scenario, mobile females might move out of a noisy area to nearby quieter habitat, but site-attached males may be unable to do so, with potentially important consequences for population demography and implications for the management of noise (Husté et al. 2006). Using only knowledge of short-term responses may also have detrimental consequences if, for instance, individuals habituate to stimuli over time and can compensate for initial impacts; mitigation measures required initially to avoid acute effects on survival (Simpson et al. 2016) might subsequently become unnecessary, and their continued implementation could be conservative. By contrast, there may be declines in growth and fitness consequences for individuals inhabiting areas exposed to chronic noise, even if no acute changes in behavior or physiology were initially displayed (Slabbekoorn et al. 2010).

In general, there is a need to determine both the spatial and temporal scale of noise impact, including the establishment of noise-exposure threshold levels (those at which different biological responses are predicted; Farcas et al. 2016). Incorporation of intraspecific variation into these threshold levels will improve their accuracy; the result might then be multiple spatial zones where different subsets of a population are likely to be affected. Additionally, impacts of noise during the breeding season might be especially detrimental, due to the increased fitness costs associated with reproductive failure (Nedelec et al. 2017b), and so noise mitigation during this period might have a greater population-level impact than at other times of year. As an existing conservation example, there is season-specific legislation on noise pollution with respect to marine mammal movement and behavioral patterns (Merchant et al. 2018; Pine et al. 2019). For instance, the *Be Whale Wise* regulations are a set of guidelines, developed collaboratively by government agencies, nonprofit organizations and local stakeholders, for boat users in the Salish Sea (USA and Canada). These guidelines instruct users to take extra care to reduce vessel noise in the area between May and September, which is the breeding and pupping seasons for marine mammals (Be Whale Wise 2019). Overall, the aim should be to use information about intraspecific variation in noise responses to enhance the effectiveness of mitigation strategies.

## CONCLUSIONS

Over the last 10–15 years, a rapidly burgeoning literature has provided substantial evidence that nonhuman animals from a wide range of taxa and ecosystems are detrimentally affected by anthropogenic noise (Morley et al. 2014; Shannon et al. 2015; Kunc et al. 2016). While there is undoubted value in continuing to extend the geographic and taxonomic range of sampling (Shannon et al. 2015), there is also a need to expand the scope of studies. We argue that including greater consideration of intraspecific variation in responses would represent a profitable and important expansion in this regard. Doing so will generate a more complete and realistic understanding across all levels of biological organization, helping to prevent misinterpretations that can lead to over- or under-estimation of the impacts of noise exposure. While it is well-recognized that care should be taken when extrapolating results between species, as responses may differ substantially (Shannon et al. 2015; Kunc et al. 2016), similar caution should be applied within species. Moreover, inclusion of intraspecific response variation in studies will enable better translation into suitable and effective management recommendations and actions. With continued urbanization, energy-generation and transport-network expansion in a range of different ecosystems, it is likely that anthropogenic noise will become ever more prevalent across the globe. To understand its true impact and to mitigate its effects, we must pay careful attention to the variation that exists within populations and species.

## Supplementary Material

arz114_suppl_Supplementary_Material
