# Supplementary material for: Causes and consequences of intraspecific variation in animal responses to anthropogenic noise
Source: Behav Ecol. 2019 Jul 1;30(6):1501–11. doi: 10.1093/beheco/arz114 (PMC6838653; doi:10.1093/beheco/arz114)
Supplement: arz114_suppl_Supplementary_Material [file arz114_suppl_supplementary_material.docx]

**Supplementary information**

**Methodology - Systematic literature review**

We conducted a systematic review of the peer-reviewed literature; see Figure S1 for a schematic of our methodology. Initially, we used “ANTHROPOGENIC NOISE” OR “ACOUSTIC DISTURBANC*” OR “NOISE POLLUT*” OR “MAN-MADE NOIS*” as search terms in Thompson’s *ISI Web of Science*, to find potentially relevant papers published between 1900 and 2018 (n = 3,309 records). In addition, we conducted a search in *Web of Science* using the same methodology as Shannon *et al*. (2015)—the most comprehensive recent review on the impacts of anthropogenic noise on non-human animals—but for 2013–2018 (the years since their search), which produced 1,516 records. Alongside these database searches, we included the 242 papers cited in the Shannon *et al*. (2015) review and papers that have cited Shannon *et al*. (2015) (n = 69; as of 05/10/2018 in *Web of Science*) in our initial list. Subsequent selection steps followed the Prisma protocol for systematic reviews (Moher et al. 2009). We used JabRef (http://www.jabref.org/) bibliography reference manager to check the combined records (n = 5,136) for duplicates, resulting in 4,558 unique records for the screening phase. We then screened papers for those that focussed on the impact of anthropogenic noise on non-human animals, based on a review of the title and abstract. Our criteria for noise selection were that studies had used real anthropogenic noise sources, or their playback equivalents, or synthetic noise; we excluded studies using pure tones as an acoustic disturbance as these are generally used in hearing assessments or as stimuli for training. We also excluded reviews, modelling studies (predicting risk factors from known hearing thresholds), experimental studies with a sample size of 1, and observational studies where impacts of noise were suggested but noise measurements not taken. Overall, the screening phase retained 589 records, which we then fully assessed to identify observational and experimental studies testing intraspecific variation in non-human animal responses to anthropogenic noise.

**4,825** records identified through database searches

**311** additional records

(papers from & those that cite Shannon *et al*. 2015)

Identification

**4,558** records after duplicates removed

Screening

**4,558**

records screened

**3,969**

records excluded

**589** full-text articles

assessed for eligibility

**524** full-text articles excluded (don’t explicitly test intraspecific variation)

Eligibility

**51** experimental studies

(explicitly testing

intraspecific variation)

(see Table S1)

Included

**14** observational studies excluded (explicitly testing intraspecific variation)

(see Table S2)

**Supplementary Figure S1. PRIMSA flow diagram outlining the systematic literature review process.**

**Methodology - Effect size calculations**

Where possible for studies in Table S1, we calculated standardised effect sizes (*Hedge’s g*) for each treatment group, correcting for small sample size bias, using the compute.es package in R (R Core Team, 2016; www.R-project.org). Effect sizes were calculated for all studies where the means, sample sizes and standard error or standard deviation were presented or were accessible from the raw data, or where independent statistical tests per condition were available. Effect sizes for studies using a within-subjects design were only calculated if the correlation between the repeated measures were accessible (Borenstein 2009). In addition, WebPlotDigitizer (https://automeris.io/WebPlotDigitizer/) was used to extract these metrics from paper figures when they were not included in the text, with such software shown to provide high accuracy and validity when extracting data from plots (Drevon et al. 2017). For instances where multiple response measures considering intraspecific variation were shown to be significant or where there was no effect found in any of those response measures, a single example was used (six out of 15; 40%). Further, where possible, we determined an overall (composite) effect size of each intrinsic characteristic and extrinsic factor for each paper, to enable quantitative assessment of the relative potential importance of each source of intraspecific variation. Composite effect sizes were determined following the methods of Borenstein et al. (2009) on computing combined effects within individual studies (see Figure 2b).

**Table S1.** **Experimental studies investigating intraspecific variation in responses to anthropogenic noise as a consequence of** **(a)** **intrinsic characteristics and (b) extrinsic factors, as reported from the primary research papers.** Examples use either real or playback of anthropogenic noise, or playback of either pink, white or brown noise as an acoustic disturbance. Effect size statistics are in bold and represent the standardised mean difference, corrected for small sample size bias, and the corresponding confidence intervals as calculated from available information. The direction for each individual effect size are presented so that positive integers represent anthropogenic noise increasing the response measure, and negative integers causing a decrease in response.

| Intraspecific variation | Examples | References |
| --- | --- | --- |
| 1. Intrinsic characteristics | | |
| Body size/ Age | Hybrid striped bass (white bass (*Morone chrysops*) x striped bass (*Morone saxatilis*)) of larger size suffered more severe internal injuries than smaller individuals when exposed to pile-driving noise playback. | (Casper et al. 2013) |
|  | Shore crabs (*Carcinus maenas*) of larger size had disproportionately higher oxygen-consumption rates than smaller conspecifics in response to ship-noise playback compared to ambient-sound controls. | (Wale et al. 2013) |
|  | Younger birds of various species showed greater avoidance than older individuals when exposed to traffic-noise playback, with the capture ratio of young/adult birds declining in locations exposed to traffic noise. | (McClure et al. 2017) |
|  | Zebra finch (*Taenopygia guttata*) post-fledglings had a greater reduction in telomere length (**Hedge’s *g*: -0.68; CI: -1 – -0.35**) than pre-fledglings (**Hedge’s *g*: -0.04; CI: -0.32–0.23**) when exposed to traffic-noise playback. | (Dorado-Correa et al. 2018) |
|  | Seabass (Dicentrarchus labrax) of smaller size startled in response to playback of pile-driving noise at a lower sound level than larger conspecifics. | (Kastelein et al. 2017) |
|  | California sea lions (*Zalophus californianus*) exposed to mid-frequency sonar exhibited a dose-response relationship with increasing sound levels; however, the probability of response at lower exposure levels declined with the removal of individuals under the age of two years. | (Houser et al. 2013) |
| Body condition | European eels (*Anguilla anguilla*) in poorer condition exhibited a reduced response to a simulated predatory strike when played ship noise compared to ambient sound **(Hedge’s *g*: -0.64; CI: -1.28– -0.01)**, whereas better-quality individuals showed no such noise effect **(Hedge’s *g*: -0.18; CI: -0.79–0.42)**. | (Purser et al. 2016) |
|  | Zebra finch (*Taeniopygia guttata*) offspring survival rates when exposed to chronic traffic noise tended to increase when the maternal baseline corticosterone levels were higher, whereas no similar trend was found in survival rate under no noise conditions. | (Potvin and Macdougall-Shackleton 2015) |
| Sex | Daffodil cichlid (*Neolamprologus pulcher*) dominant males removed sand from their nests fewer times during boat-noise playback than in ambient-sound conditions, whereas dominant females showed no sound-treatment difference in sand-digging frequency. | (Bruintjes and Radford 2013) |
|  | Female wild mice (*Mus musculus*) did not differ in their corticosterone levels when exposed to low-frequency mining noise compared to control conditions, whereas males had higher corticosterone levels in mining-noise conditions compared to control conditions | (Mancera et al. 2017) |
|  | Spiny lobster (*Jasus edwardsii*) females and males both had lower total haemocyte counts after being exposed to sound from a seismic air gun compared to ambient controls. | (Fitzgibbon et al. 2017) |
|  | Tree swallow (*Tachycineta bicolor*) female settlement was delayed by an increase in background traffic noise, whereas there was no noise-induced delay in male settlement. | (Injaian et al. 2018) |
|  | Greater sage-grouse (*Centrocercus urophasianus*) male abundance declined on leks exposed to drilling or traffic noise compared to ambient control leks, with weaker, but similar, effects of noise playback on female abundance. | (Blickley et al. 2012) |
|  | Rat (*Rattus sp.*) males exposed to vibrational noise spent less time sitting post-noise exposure compared to control rats, whereas females spent more time sitting post-noise exposure compared to the control group. | (Avaliani et al. 2018) |
|  | Great tit (*Parus major)* males with low exploratory scores avoided the white-noise playback more than males with high exploratory scores, this effect was reversed in females. | (Naguib et al. 2013) |
|  | Mediterranean spiny lobster (*Palinurus elephas*) males and females both had elevated expression levels of the Hsp70 protein (% integrated density value) when exposed to boat-noise playback compared to control conditions. | (Filiciotto et al. 2014) |
| Personality | Great tits (*Parus major*) characterised with high exploratory scores had a lower latency time to first nestbox visit than birds with low exploratory scores during white-noise playback. | (Naguib et al. 2013) |

| (b) Extrinsic factors | | | |
| --- | --- | --- | --- |
| Context | Daffodil cichlids (*Neolamprologus pulcher*) showed no difference in anti-predator defence behaviours when exposed to boat-noise and ambient-sound playback when they had eggs in their nest (**Hedge’s *g*: -0.29; CI: -1.15–0.57)**, but there was less defensive behaviour during boat-noise playback than ambient-sound playback in the absence of eggs (**Hedge’s *g*: -0.79; CI: -1.54– -0.03)**. | (Bruintjes and Radford 2013) |  |
|  | European sea bass (*Dicentrarchus labrax*) exposed to playback of impulsive noise (filtered brown noise) showed a greater increase in group cohesion at night than during the day. | (Neo et al. 2018) |  |
|  | Largemouth bass (*Micropterus salmoides*) adults showed no significant difference in the number of turns over their nest (vigilance behaviour) during exposure to motorboat noise compared to the pre-treatment period, a consistent response was shown across three offspring developmental stages. | (Maxwell et al. 2018) |  |
|  | Australian snapper (*Pagrus auratus*) inhabiting an open (unprotected) habitat decreased feeding activity and displayed avoidance behaviours during motorboat passes compared to pre- and post-sound periods, but these effects were absent in fish inhabiting a protected area. | (Mensinger et al. 2018) |  |
|  | Gobies (*Gobius cruentatus)* were more submissive towards an intruder during boat-noise playback compared to a silent control when resident on a territory, whereas individuals acting as an intruder during a similar territorial encounter spent less time displaying submissive behaviours in the noise treatment compared to the silent control. | (Sebastianutto et al. 2011) |  |
|  | House wren (*Troglodytes aedon*) males that were part of a pair displayed a higher peak frequency in their songs than unpaired males during ‘pink’ noise exposure. | (Grabarczyk et al. 2018) |  |
|  | Atlantic herring (*Clupea harengus*) shoals of low density showed stronger avoidance behaviours than high-density shoals in response to vessel-noise playback. | (Handegard et al. 2015) |  |
|  | House wren (Troglodytes aedon) adults inhabiting a rural environment had an increase in corticosterone following exposure to traffic-noise playback **(Hedge’s g: 2.16; CI: 0.75–3.57)**, whereas urban conspecifics exhibited no such response to the noise exposure **Hedge’s g: 0.85; CI: -0.09–1.79)**. | (Davies et al. 2017) |  |
|  | Tree frogs (*Hyla arborea*) singing alone did not adjust their singing bout duration during exposure to traffic-noise playback **(Hedge’s *g*: -0.46; CI: -1.08–0.16)**, whereas a noise-induced change in calling activity was observed for tree frogs singing in a chorus **(Hedge’s *g*: -1.09; CI: -1.9– -0.28)**. | (Lengagne 2008) |  |
|  | Perch (*Perca fluviatilis*) in single-species enclosures reduced feeding attempts during exposure to motorboat noise, whereas no effect of motorboat-noise exposure was evident for perch in mixed-species enclosures with roach (*Rutilus rutilus*). | (Magnhagen et al. 2017) |  |
|  | Blue whales (*Balaenotera musculus*) that were deep-feeding were affected by exposure to mid-frequency sonar, whereas whales in other behavioural states showed less response. | (Goldbogen et al. 2013) |  |
|  | Mediterranean spiny lobsters (*Palinurus elephas*) in groups of four increased both their distance moved and velocity when exposed to boat-noise playback compared to control groups; no such increases were apparent when tested alone. | (Filiciotto et al. 2014) |  |
|  | White-crowned sparrow (*Zonotrichia leucophrys*) males in urban areas decreased the bandwidth of their song when exposed to playback of experimental noise compared to before noise playback, whereas rural males did not adjust their song bandwidth. | (Gentry et al. 2017) |  |
| Repeated exposure | Ambon damselfish (*Pomacentrus amboinensis*) exposed to real motorboat noise showed immediate reductions in boldness relative to ambient-sound controls (**Hedge’s *g*: -1.36; CI: -2.07– -0.66**), but fish returned to pre-boat exposure behaviours within the 20-min trial (**Hedge’s *g*: -0.02; CI:-0.65–0.62**). | (Holmes et al. 2017) |  |
|  | European sea bass (*Dicentrarchus labrax*) exposed to impulsive noise (filtered brown noise) swam faster, deeper and in tighter shoals compared to pre-exposure baseline levels, but recovered within the 60-min exposure period. | (Neo et al. 2015) |  |
|  | Lined seahorses (*Hippocampus erectus*) exposed to high ambient-sound levels from aquarium machinery initially made more tail adjustments whilst stationary than those in quiet control tanks (**Hedge’s *g*: 0.79; CI: -0.32–1.9**), but this response difference diminished after three weeks (**Hedge’s *g***: **0.05; CI: -0.96–1.06**). | (Anderson et al. 2011) |  |
|  | Threespot dascyllus (*Dascyllus trimaculatus*) exposed to motorboat-noise playback initially exhibited elevated ventilation rates compared to ambient controls (**Hedge’s *g*: 1.68; CI: 0.96–2.39**), but these responses lessened after one week of repeated exposure (**Hedge’s *g*: 0.51; CI: -0.09–1.11**). | (Nedelec et al. 2016) |  |
|  | Greater mouse-eared bats (*Myotis myotis*) displayed a higher proportion of responses when first exposed to short-term traffic-noise playback compared to a silent control, but the proportion of responses was reduced when bats were exposed to a second exposure. | (Luo et al. 2014) |  |
|  | California sea lions (*Zalophus californianus*) exposed to mid-frequency sonar exhibited a dose-response relationship with increasing sound levels, and responses did not change over repeated exposures of the noise stimulus. | (Houser et al. 2013) |  |
|  | Bottlenose dolphins (*Tursiops truncatus*) exposed to mid-frequency sonar exhibited a dose-response relationship with increasing sound levels, however the probability of response at sound levels below 160 dB re 1 μPa decreased with repeated exposure of the noise stimulus. | (Houser et al. 2013) |  |
|  | American black ducks (*Anus rubripes*) reacted (alert & fleeing responses) to simulated aircraft overflight noise on first exposure, but the proportion of occasions that the birds reacted declined with repeated exposure to the noise stimulus. | (Conomy et al. 1998) |  |
|  | Killer whales (*Orcinus orca*) increased their distance from the vessel when first exposed to an acoustic harassment device that was turned on rather than turned off, but the distance from the vessel no longer changed when the device was on vs off during successive exposures. | (Tixier et al. 2015) |  |
|  | Atlantic bottlenose dolphins (*Tursiops truncatus*) showed reduced target-detection performance when first exposed to vibratory pile-driving noise playback compared to control periods, but no change in target-detection performance was evident by the second replication. | (Branstetter et al. 2018) |  |
|  | Perch (*Perca fluviatilis*) in single-species enclosures made fewer feeding attempts during noise exposure compared to control trials, but over multiple days the number of feeding attempts increased in both treatments. | (Magnhagen et al. 2017) |  |
|  | European sea bass (*Dicentrarchus labrax*) exposed to playback of filtered brown noise displayed increases in swimming depth, but these changes declined with repeated exposure over eight trials. | (Neo et al. 2018) |  |
|  | Shore crabs (*Carcinus maenas*) exposed to ship-noise playback had a significantly higher oxygen-consumption rate compared to ambient controls, but responses did not change during repeated exposure over 16 days, whereas oxygen-consumption increased over the duration of multiple trials for the ambient control crabs. | (Wale et al. 2013) |  |
|  | European seabass (*Dicentrarchus labrax*) exposed to filtered brown noise of varying temporal structure increased their swimming depth and group cohesion at the onset of sound exposure, but behavioural measures returned to baseline levels within the 30-min exposure period. | (Neo et al. 2014) |  |
|  | European seabass (*Dicentrarchus labrax*) exposed to filtered brown noise increased their swimming depth at the onset of sound exposure, but returned to baseline behaviour within the 60-min exposure period. | (Neo et al. 2016) |  |
|  | European perch *(Perca fluviatilis)* exposed to boat disturbance increased swimming activity during the first hour of exposure, but this response declined to pre-exposure levels in the next three hours of boat disturbance*.* | (Jacobsen et al. 2014) |  |
| Prior experience | Mountain chickadee (*Poecile gambeli*) males from characteristically noisy areas responded to white-noise playback (with a frequency spectrum of traffic noise) by singing at higher frequencies and using fewer songs from their lower bandwidth repertoire, whereas individuals from quieter areas showed opposite responses. | (LaZerte et al. 2016) |  |
|  | Mountain chickadee (*Poecile gambeli*) males from characteristically noisy areas exposed to experimental noise playback (white noise with a frequency spectrum of traffic noise) switched their vocalisation type to use more songs than calls, whereas males from quiet areas used relatively more calls. | (LaZerte et al. 2017) |  |
|  | Ezo brown frogs (*Rana pirica*) from characteristically noisy sites displayed faster initiation of phonotaxis and a stronger avoidance response under non-overlapping and overlapping vehicle noise respectively, compared to frogs from quiet sites. | (Senzaki et al. 2018) |  |
|  | Naïve European sea bass (*Dicentrarchus labrax*) exposed to pile-driving noise had significantly elevated opercular beat rates compared to ambient-control fish (**Hedge’s *g*: 0.62; CI: 0.1–1.14**); fish exposed to 12 weeks of pile-driving noise displayed reductions in response relative to ambient-control fish (**Hedge’s *g*: 0.16; CI: -0.36–0.67**), | (Radford et al. 2016) |  |
|  | Field cricket (*Teleogryllus oceanicus*) females reared in masking traffic noise took longer to start searching and to reach a speaker simulating a singing male compared to crickets reared in silent conditions irrespective of the acoustic exposure during the testing period (masking noise, non-masking noise, silent control). | (Gurule-Small and Tinghitella 2018) |  |
|  | Golden-cheeked warblers (*Setophaga chrysoparia*) from characteristically noisy sites and those from quiet sites both showed no difference in behavioural responses when exposed to playback of construction noise compared to control playbacks. | (Long et al. 2017) |  |
|  | Golden-cheeked warblers (*Setophaga chrysoparia*) from characteristically noisy sites and those from quiet sites were both more likely to respond to construction-noise playback than a silent control. | (Long et al. 2017) |  |
|  | *Cynotilapia zebroides* males from lower-disturbance sites displayed an increase in oxygen-consumption rate compared to ambient controls when exposed to motorboat noise (**Hedge’s *g*: 0.51; CI: 0.18–0.85**), whereas fish from higher-disturbance sites showed no difference from ambient controls  (**Hedge’s *g*:** **0.15; CI: -0.18–0.48**). | (Harding et al. 2018) |  |
| Multiple stressors | Damselfish (*Pomacentrus wardi*) and dottyback (*Pseudochromis fuscus*) predator–prey interactions were affected when exposed to elevated CO_2_ and motorboat-noise playback both in isolation and when combined compared to ambient conditions; e.g. under present-day CO_2_, predator attack speed was reduced when exposed to motorboat-noise playback compared to ambient controls (**Hedge’s *g*: -0.62; CI: -1.31–0.07**). Under high CO_2_ conditions, fish exposed to noise showed no difference from ambient controls (**Hedge’s *g*: -0.07; CI: -0.72–0.59**). | (McCormick et al. 2018) |  |
|  | European sea bass (*Dicentrarchus labrax*) exposed to pile-driving noise playback under present-day CO_2_ conditions had increased ventilation rates compared to ambient-sound controls under equivalent CO_2_ conditions (**Hedge’s *g*: 1.51; CI: 0.84–2.17**). Under elevated CO_2_ conditions, responses to noise were comparable (**Hedge’s *g*: 1.69; CI: 1–2.37**); there was no interaction between CO_2_ and noise treatment. | (Poulton et al. 2016) |  |
|  | Zebrafish (*Danio rerio*) exposed to white noise spent more time stationary and avoided the active speaker compared to ambient conditions, whereas dim light caused a lower number of crossings between the treatment tank and escape tank, and more time spent in the upper layer of the tank. There were no interactions between sound and light on zebrafish behaviour. | (Sabet et al. 2016) |  |
|  | Frog-biting midges (*Corethrella spp.*) were collected in acoustic traps in smaller numbers when exposed to anthropogenic noise in low light levels, whereas in high-light conditions very few midges were collected irrespective of the noise exposure. | (McMahon et al. 2017) |  |

**Table S2.** **Observational studies investigating intraspecific variation in responses to anthropogenic noise as a consequence of** **intrinsic characteristics and extrinsic factors, as reported from the primary research papers.**

| Intraspecific variation | Observational Examples | References |  |
| --- | --- | --- | --- |
| Intrinsic characteristics | | | |
| Body size/ Age | Cod (*Gadus morhua*) and haddock (*Melanogrammus aeglefinus*) of larger size exhibited a greater decline in abundance and catch rate than smaller individuals when there were seismic-gun surveys. | (Engås et al. 1996) |  |
|  | Ovenbirds (*Seiurus aurocapilla*) with less experience (were younger) were more abundant at sites near compressor stations compared to quiet areas, causing a difference in age structure between noisy and control sites | (Habib et al. 2006) |  |
| Sex | A male giant panda (*Ailuropoda melanoleuca*), but not a female, in captivity displayed behavioural responses to construction noise compared to quiet days. | (Powell et al. 2006) |  |
|  | A female giant panda (*Ailuropoda melanoleuca*), but not a male, in captivity displayed elevated cortisol levels on loud days compared to quiet days. | (Owen et al. 2004) |  |
| Context | Killer whale (*Orcinus orca*) presence in a fjord system was negatively affected by exposure to sonar type, especially during periods of low-prey availability. | (Kuningas et al. 2013) |  |
|  | Bottlenose dolphins (*Tursiops truncatus*) disturbed by tour boats showed little change in maximum frequency of whistles when calves were present, but had a higher maximum frequency when calves were absent. | (Heiler et al. 2016) |  |
|  | Guiana dolphins (*Sotalia guianensis*) changed their whistling rate when exposed to high noise levels compared to quieter periods if they were feeding but not whilst engaged in social behaviours. | (Bittencourt et al. 2017) |  |
|  | Chipping sparrow (*Spizella passerina*) males whose song was characterised with lower minimum frequencies and broader bandwidths increased their minimum frequency and decreased bandwidth in response to noise, whereas those whose song had higher minimum frequencies and narrower bandwidths showed no such song adjustments in noise. | (Job et al. 2016) |  |
| Repeated  exposure | Harbour porpoises (*Phocoena phocoena*) showed a reduction in occurrence from exposure to a seismic airgun, although the level of response declined over the 10-day seismic survey period. | (Thompson et al. 2013) |  |
| Multiple stressors | Great tit (*Parus major*) nestlings exposed to high anthropogenic noise had elevated levels of haptoglobin, whereas artificial light at night and its interaction with noise had no effect on nestling physiology. | (Raap et al. 2017) |  |
|  | European blackbirds (*Turdus merula*) started their dawn chorus earlier in areas with high traffic noise, although the shift was not completely separable from the effects of ambient light at night. | (Nordt and Klenke 2013) |  |
|  | Rufous-collared sparrows (*Zonotrichia capensis*) in areas with higher daytime noise started the dawn chorus earlier compared to areas with low anthropogenic noise, whereas light pollution levels didn’t affect the onset of the dawn chorus. | (Dorado-Correa et al. 2016) |  |
|  | Five species of common European songbird sang earlier at dawn when occupying sites with high artificial light at night, whereas anthropogenic noise showed little effect on the timing of the dawn chorus for all species. | (Da Silva et al. 2014) |  |
|  | Great tit (*Parus major*) nestlings from sites characterised with varying levels of anthropogenic noise, artificial light at night, and distance from roads explained the variation in oxidative status markers. | (Casasole et al. 2017) |  |

**Table S3. Decisions to aid the design and implementation of studies considering intraspecific variation.**

| Decision topic | Explanation | | Examples of good practice | | | % of studies in Table S1 |  |
| --- | --- | --- | --- | --- | --- | --- | --- |
| Experimental design | | | | | | |  |
| Suitable controls | Suitable control treatments are required so that baseline differences between the relevant categories are established | | (Neo et al. 2018) | | | Up to 10% did not clearly report this information |  |
| Measures of fitness | Establishing impacts on fitness directly, rather than extrapolating from short-term behavioural or physiological responses | | Casper *et al.* (2013)  Potvin & Macdougall-Shackleton (2015) | | | 96% (49/51) did not measure fitness in relation to intraspecific variation |  |
| Pseudoreplication | Failure to replicate at one or more levels within an experiment (e.g. in subjects, or failure to replicate sound sources or exemplars) | | (McCormick et al. 2018) | | | 51% (18/35) used only one real-noise source or playback exemplar |  |
| Sample size | Need sufficient sample sizes for each category being considered (e.g. males and females) rather than just the overall number of subjects | | (Dorado-Correa et al. 2018) | | | 43% (19/44) had <10 individuals per category |  |
| Captive/laboratory vs field-based experiments | | | | | | | |
| Experimental control | Laboratory experiments can be tightly controlled and isolated from confounding variables, allowing determination of underlying mechanisms, which is harder to achieve in field experiments | |  | | | 47% (24/51) were captive/laboratory studies  53% (27/51) were field-based experiments  No study combined both approaches |  |
| Behavioural responses | Confined animals in laboratory experiments may display a different or narrower range of behaviours. Field-experiments allow natural behaviours to be observed | |  | | |  |  |
| Acoustic measurements | | | | | | | |
| Full characterisation of the sound field | | For aquatic studies on fish and invertebrates, this includes reporting measures from both particle-motion and sound-pressure domains. For terrestrial studies, the correct frequency weighting for the taxa needs to be applied | | (LaZerte et al. 2016; LaZerte et al. 2017; Sabet *et al*. 2016) | 38% did not report detailed acoustic information (14 out of 25 fish and aquatic invertebrate studies; 3 out of 20 terrestrial studies) | | |
| Complementary use of real noise sources and loudspeaker playback | | Loudspeaker playback isolates the noise as the stressor, free from visual disturbances and other potential confounds. However, loudspeaker use can result in sound fields that can vary considerably from those in real-world situations. Real noise sources are required for acoustic validity, but their use can be logistically challenging and minimising pseudoreplication is difficult. Complementary use of both methods would be best practice. | | (Harding *et al*. 2018) | 98% use only either loudspeaker playback or real noise sources (50 out of 51). | | |

**References**

Anderson PA, Berzins IK, Fogarty F, Hamlin HJ, Guillette LJ. 2011. Sound, stress, and seahorses: the consequences of a noisy environment to animal health. Aquaculture. 311:129–138.

Avaliani TV, Klyueva NN, Apraksina NK, Tsikunov SG. 2018. Gender-specific features of behavior and lipid spectrum in rats at a remote period after exposure to vibrational noise. J Evol Biochem Physiol. 54:123–129.

Bittencourt L, Lima IMS, Andrade LG, Carvalho RR, Bisi TL, Lailson-Brito Jr. J, Azevedo AF. 2017. Underwater noise in an impacted environment can affect Guiana dolphin communication. Mar Pollut Bull. 114:1130–1134.

Blickley JL, Blackwood D, Patricelli GL. 2012. Experimental evidence for the effects of chronic anthropogenic noise on abundance of greater sage-grouse at leks. Conserv Biol. 26:461–471.

Borenstein M. 2009. Effect sizes for continuous data. In The Handbook of Research Synthesis & Meta-Analysis. New York: Russell Sage Foundation. p. 222–235.

Borenstein M, Hedges LV, Higgins JPT, Rothstein HR. 2009. Complex data structures. In Introduction to meta-analysis. Chester, England: Wiley. p.217–223.

Branstetter BK, Bowman VF, Houser DS, Tormey M, Banks P, Finneran JJ, Jenkins K. 2018. Effects of vibratory pile driver noise on echolocation and vigilance in bottlenose dolphins (*Tursiops truncatus*). J Acoust Soc Am. 143:429–439.

Bruintjes R, Radford AN. 2013. Context-dependent impacts of anthropogenic noise on individual and social behaviour in a cooperatively breeding fish. Anim Behav. 85:1343–1349.

Casasole G, Raap T, Costantini D, AbdElgawad H, Asard H, Pinxten R, Eens M. 2017. Neither artificial light at night, anthropogenic noise nor distance from roads are associated with oxidative status of nestlings in an urban population of songbirds. Comp Biochem Physiol Part A. 210:14–21.

Casper BM, Halvorsen MB, Matthews F, Carlson TJ, Popper AN. 2013. Recovery of barotrauma injuries resulting from exposure to pile driving sound in two sizes of hybrid striped bass. PLoS One. 8:e73844.

Conomy JT, Dubovsky JA, Collazo JA, Fleming WJ. 1998. Do black ducks and wood ducks habituate to aircraft disturbance? J Wildl Manage. 62:1135–1142.

Davies S, Haddad N, Ouyang JQ. 2017. Stressful city sounds: glucocorticoid responses to experimental traffic noise are environmentally dependent. Biol Lett. 13:20170276.

Dorado-Correa AM, Rodriguez-Rocha M, Brumm H. 2016. Anthropogenic noise, but not artificial light levels predicts song behaviour in an equatorial bird. R Soc Open Sci. 3:160231.

Dorado-Correa AM, Zollinger SA, Heidinger B, Brumm H. 2018. Timing matters: traffic noise accelerates telomere loss rate differently across developmental stages. Front Zool. 15:29.

Drevon D, Fursa SR, Malcolm AL. 2017. Intercoder reliability and validity of webplotdigitizer in extracting graphed data. Behav Modif.41:323–339.

Engås A, Løkkeborg S, Ona E, Soldal AV. 1996. Effects of seismic shooting on local abundance and catch rates of cod (*Gadus morhua*) and haddock (*Melanogrammus aeglefinus*). Can J Fish Aquat Sci. 53:2238–2249.

Filiciotto F, Vazzana M, Celi M, Maccarrone V, Ceraulo M, Buffa G, Di Stefano V, Mazzola S, Buscaino G. 2014. Behavioural and biochemical stress responses of *Palinurus elephas* after exposure to boat noise pollution in tank. Mar Pollut Bull. 84:104–114.

Fitzgibbon QP, Day RD, McCauley RD, Simon CJ, Semmens JM. 2017. The impact of seismic air gun exposure on the haemolymph physiology and nutritional condition condition of spiny lobster, *Jasus edwardsii*. Mar Pollut Bull. 125:146–156.

Gentry KE, Derryberry EP, Danner RM, Danner JE, Luther DA. 2017. Immediate signaling flexibility in response to experimental noise in urban, but not rural, white-crowned sparrows. Ecosphere. 8:e01916.

Goldbogen JA, Southall BL, DeRuiter SL, Calambokidis J, Friedlaender AS, Hazen EL, Falcone EA, Schorr GS, Douglas A, Moretti DJ, et al. 2013. Blue whales respond to simulated mid-frequency military sonar. Proc R Soc B. 280:20130657.

Grabarczyk EE, Pipkin MA, Vonhof MJ, Gill SA. 2018. When to change your tune? Unpaired and paired male house wrens respond differently to anthropogenic noise. J Ecoacoust. 2:#LHGRVC.

Gurule-Small GA, Tinghitella RM. 2018. Developmental experience with anthropogenic noise hinders adult mate location in an acoustically signalling invertebrate. Biol Lett. 14:20170714.

Habib L, Bayne EM, Boutin S. 2006. Chronic industrial noise affects pairing success and age structure of ovenbirds *Seiurus aurocapilla*. J Appl Ecol. 44:176–184.

Handegard NO, De Robertis A, Rieucau G, Boswell K, Macaulay GJ. 2015. The reaction of a captive herring school to playbacks of a noise-reduced and a conventional research vessel. Can J Fish Aquat Sci. 72:491–499.

Harding HR, Gordon TAC, Hsuan RE, Mackaness ACE, Radford AN, Simpson SD. 2018. Fish in habitats with higher motorboat disturbance show reduced sensitivity to motorboat noise. Biol Lett. 14:20180441.

Heiler J, Elwen SH, Kriesell HJ, Gridley T. 2016. Changes in bottlenose dolphin whistle parameters related to vessel presence, surface behaviour and group composition. Anim Behav. 117:167–177.

Holmes LJ, McWilliam J, Ferrari MCO, McCormick MI. 2017. Juvenile damselfish are affected but desensitize to small motor boat noise. J Exp Mar Bio Ecol. 494:63–68.

Houser DS, Martin SW, Finneran JJ. 2013. Behavioral responses of California sea lions to mid-frequency (3250-3450 Hz) sonar signals. Mar Environ Res. 92:268–278.

Houser DS, Martin SW, Finneran JJ. 2013. Exposure amplitude and repetition affect bottlenose dolphin behavioral responses to simulated mid-frequency sonar signals. J Exp Mar Bio Ecol. 443:123–133.

Injaian AS, Poon LY, Patricelli GL. 2018. Effects of experimental anthropogenic noise on avian settlement patterns and reproductive success. Behav Ecol. 29:1181–1189.

Jacobsen L, Baktoft H, Jepsen N, Aarestrup K, Berg S, Skov C. 2014. Effect of boat noise and angling on lake fish behaviour. J Fish Biol. 84:1768–1780.

Job JR, Kohler SL, Gill SA. 2016. Song adjustments by an open habitat bird to anthropogenic noise, urban structure, and vegetation. Behav Ecol. 27:1734–1744.

Kastelein RA, Jennings N, Kommeren A, Helder-Hoek L, Schop J. 2017. Acoustic dose-behavioral response relationship in sea bass (*Dicentrarchus labrax*) exposed to playbacks of pile driving sounds. Mar Environ Res. 130:315–324.

Kuningas S, Kvadsheim PH, Lam F-PA, Miller PJO. 2013. Killer whale presence in relation to naval sonar activity and prey abundance in northern Norway. ICES J Mar Sci. 70:1287–1293.

LaZerte SE, Otter KA, Slabbekoorn H. 2017. Mountain chickadees adjust songs, calls and chorus composition with increasing ambient and experimental anthropogenic noise. Urban Ecosyst. 20:989–1000.

LaZerte SE, Slabbekoorn H, Otter KA. 2016. Learning to cope: vocal adjustment to urban noise is correlated with prior experience in black-capped chickadees. Proc R Soc B. 283:20161058.

Lengagne T. 2008. Traffic noise affects communication behaviour in a breeding anuran, *Hyla arborea*. Biol Conserv. 141:2023–2031.

Long AM, Colon MR, Bosman JL, McFarland TM, Locatelli AJ, Stewart LR, Mathewson HA, Newnam JC, Morrison ML. 2017. Effects of road construction noise on golden-cheeked warblers: an update. Wildl Soc Bull. 41:240–248.

Long AM, Colon MR, Bosman JL, Robinson DH, Pruett HL, McFarland TM, Mathewson HA, Szewczak JM, Newnam JC, Morrison ML. 2017. A before-after control-impact assessment to understand the potential impacts of highway construction noise and activity on an endangered songbird. Ecol Evol. 7:379–389.

Luo J, Clarin B-M, Borissov IM, Siemers BM. 2014. Are torpid bats immune to anthropogenic noise? J Exp Biol. 217:1072–1078.

Magnhagen C, Johansson K, Sigray P. 2017. Effects of motorboat noise on foraging behaviour in Eurasian perch and roach: a field experiment. Mar Ecol Prog Ser. 564:115–125.

Mancera KF, Lisle A, Allavena R, Phillips CJC. 2017. The effects of mining machinery noise of different frequencies on the behaviour, faecal corticosterone and tissue morphology of wild mice (*Mus musculus*). Appl Anim Behav Sci. 197:81–89.

Maxwell RJ, Zolderdo AJ, de Bruijn R, Brownscombe JW, Staaterman E, Gallagher AJ, Cooke SJ. 2018. Does motor noise from recreational boats alter parental care behaviour of a nesting freshwater fish? Aquat Conserv Freshw Ecosyst. 28:969–978.

McClure CJW, Ware HE, Carlisle JD, Barber JR. 2017. Noise from a phantom road experiment alters the age structure of a community of migrating birds. Anim Conserv. 20:164–172.

McCormick MI, Watson S-A, Simpson SD, Allan BJM. 2018. Effect of elevated CO_2_ and small boat noise on the kinematics of predator–prey interactions. Proc R Soc B. 285:20172650.

McMahon TA, Rohr JR, Bernal XE. 2017. Light and noise pollution interact to disrupt interspecific interactions. Ecology. 98:1290–1299.

Mensinger AF, Putland RL, Radford CA. 2018. The effect of motorboat sound on Australian snapper *Pagrus auratus* inside and outside a marine reserve. Ecol Evol. 8:6438–6448.

Moher D, Liberati A, Tetzlaff J, Altman DG. 2009. Preferred reporting items for systematic reviews and meta-analyses: The PRISMA Statement. Ann Intern Med. 151:264–269.

Naguib M, van Oers K, Braakhuis A, Griffioen M, de Goede P, Waas JR. 2013. Noise annoys: effects of noise on breeding great tits depend on personality but not on noise characteristics. Anim Behav. 85:949–956.

Nedelec SL, Mills SC, Lecchini D, Nedelec B, Simpson SD, Radford AN. 2016. Repeated exposure to noise increases tolerance in a coral reef fish. Environ Pollut. 216:428–436.

Neo YY, Hubert J, Bolle L, Winter HV, ten Cate C, Slabbekoorn H. 2016. Sound exposure changes European seabass behaviour in a large outdoor floating pen: effects of temporal structure and a ramp-up procedure. Environ Pollut. 214:26–34.

Neo YY, Hubert J, Bolle LJ, Winter H V, Slabbekoorn H. 2018. European seabass respond more strongly to noise exposure at night and habituate over repeated trials of sound exposure. Environ Pollut. 239:367–374.

Neo YY, Seitz J, Kastelein RA, Winter HV, ten Cate C, Slabbekoorn H. 2014. Temporal structure of sound affects behavioural recovery from noise impact in European seabass. Biol Conserv. 178:65–73.

Neo YY, Ufkes E, Kastelein RA, Winter HV, ten Cate C, Slabbekoorn H. 2015. Impulsive sounds change European seabass swimming patterns: Influence of pulse repetition interval. Mar Pollut Bull. 97:111–117.

Nordt A, Klenke R. 2013. Sleepless in town - drivers of the temporal shift in dawn song in urban European blackbirds. PLoS One. 8:e71476.

Owen MA, Swaisgood RR, Czekala NM, Steinman K, Lindburg DG. 2004. Monitoring stress in captive giant pandas (*Ailuropoda melanoleuca*): Behavioral and hormonal responses to ambient noise. Zoo Biol. 23:147–164.

Potvin DA, Macdougall-Shackleton SA. 2015. Traffic noise affects embryo mortality and nestling growth rates in captive zebra finches. J Exp Zool. 323:722–730.

Poulton DA, Porteus CS, Simpson SD. 2016. Combined impacts of elevated CO_2_ and anthropogenic noise on European sea bass (*Dicentrarchus labrax*). ICES J Mar Sci. 74:1230–1236.

Powell DM, Carlstead K, Tarou LR, Brown JL, Monfort SL. 2006. Effects of construction noise on behavior and cortisol levels in a pair of captive giant pandas (*Ailuropoda melanoleuca*). Zoo Biol. 25:391–408.

Purser J, Bruintjes R, Simpson SD, Radford AN. 2016. Condition-dependent physiological and behavioural responses to anthropogenic noise. Physiol Behav. 155:157–161.

Raap T, Pinxten R, Casasole G, Dehnhard N, Eens M. 2017. Ambient anthropogenic noise but not light is associated with the ecophysiology of free-living songbird nestlings. Sci Rep. 7:2754.

Radford AN, Lèbre L, Lecaillon G, Nedelec SL, Simpson SD. 2016. Repeated exposure reduces the response to impulsive noise in European seabass. Glob Chang Biol. 22:3349–3360.

Sabet SS, Van Dooren D, Slabbekoorn H. 2016. Son et lumiere: sound and light effects on spatial distribution and swimming behavior in captive zebrafish. Environ Pollut. 212:480–488.

Sebastianutto L, Picciulin M, Costantini M, Ferrero EA. 2011. How boat noise affects an ecologically crucial behaviour: the case of territoriality in *Gobius cruentatus* (Gobiidae). Environ Biol Fish. 92:207–215.

Senzaki M, Kadoya T, Francis CD, Ishiyama N, Nakamura F. 2018. Suffering in receivers: negative effects of noise persist regardless of experience in female anurans. Funct Ecol. 32:2054–2064.

Shannon G, Mckenna MF, Angeloni LM, Crooks KR, Fristrup KM, Brown E, Warner KA, Nelson MD, White C, Briggs J, et al. 2015. A synthesis of two decades of research documenting the effects of noise on wildlife. Biol Rev. 91:982–1005.

Da Silva A, Samplonius JM, Schlicht E, Valcu M, Kempenaers B. 2014. Artificial night lighting rather than traffic noise affects the daily timing of dawn and dusk singing in common European songbirds. Behav Ecol. 25:1037–1047.

Thompson PM, Brookes KL, Graham IM, Barton TR, Needham K, Bradbury G, Merchant ND. 2013. Short-term disturbance by a commercial two-dimensional seismic survey does not lead to long-term displacement of harbour porpoises. Proc R Soc B. 280:20132001.

Tixier P, Gasco N, Duhamel G, Guinet C. 2015. Habituation to an acoustic harassment device (AHD) by killer whales depredating demersal longlines. ICES J Mar Sci. 72:1673–1681.

Wale MA, Simpson SD, Radford AN. 2013. Size-dependent physiological responses of shore crabs to single and repeated playback of ship noise. Biol Lett. 9:20121194.
